# Cystic Odontoma in a Patient with Hodgkin's Lymphoma

**DOI:** 10.1155/2015/292819

**Published:** 2015-11-05

**Authors:** Victor Costa, Adriana Rocha Caris, Jorge Esquiche León, Carolina Judica Ramos, Vaneska Jardini, Estela Kaminagakura

**Affiliations:** ^1^Bioscience and Oral Diagnosis Department, Institute of Science and Technology, Universidade Estadual Paulista (UNESP), School of Dentistry, 12247-004 São José dos Campos, SP, Brazil; ^2^Oral Pathology Department, Ribeirão Preto Dental School, University of São Paulo, 14040-900 Ribeirão Preto, SP, Brazil; ^3^Pediatric Dentistry Department, Institute of Science and Technology, Universidade Estadual Paulista (UNESP), School of Dentistry, 12247-004 São José dos Campos, SP, Brazil; ^4^Pediatric Oncology, “Fabiana Macêdo de Moraes” Children Treatment Center, 12244-010 São José dos Campos, SP, Brazil

## Abstract

Cystic odontoma is a rare entity, which is characterized by the association of a cyst with complex/compound odontoma. The aim of this study was to report the case of a 5-year-old male patient diagnosed previously with Hodgkin's lymphoma and treated successfully with chemotherapy and radiotherapy, who developed a mandibular odontogenic lesion. Physical examination revealed a swelling on the right side of the mandible. Radiographically, a well-defined radiolucent area surrounded by radiopaque material was observed. An incisional biopsy was performed and microscopic analysis showed a cystic lesion consisting of an atrophic epithelium comprising 2-3 cell layers and the absence of inflammation in the cystic capsule. The cyst was decompressed and the lesion was removed after 3 months of follow-up. Microscopic analysis of the surgical specimen showed a cystic hyperplastic epithelium surrounded by an intense chronic inflammatory cell infiltrate, which was in close contact with mineralized tissue resembling dentin and cementum. The final diagnosis was cystic odontoma. Since chemotherapy can affect the growth and development of infant teeth, a relationship between chemotherapy-associated adverse events and cystic odontoma is suggested in the present case.

## 1. Introduction

Odontomas are one of the most common odontogenic tumors of the jaw bones [1]. These tumors are classified as complex and compound, their etiology is unknown, and they do not show sex predilection [1]. Microscopically, odontomas consist of enamel, dentin, variable quantities of cementum, and pulp tissue [2]. The compound type is the most common and is mainly diagnosed in the anterior region of the maxilla of children and adolescents [1]. This type consists of tooth-like structures or denticles arranged on a fibrous stroma [1]. The complex type also affects young adults and occurs more frequently in the posterior region of the mandible [1]. It consists of irregular dentin, cementum, and enamel masses without a defined dental morphology [1]. Odontomas can cause cystic degeneration, although this is considered to be a rare phenomenon [3–5].

Complex cystic odontomas are usually detected during clinical examination due to swelling, absence of a tooth, pain, or infection and normally affect the lower molar region [3]. Microscopically, complex cystic odontomas are characterized by the presence of stratified squamous epithelium, similar to the dental follicle, associated with an odontoma [3–5]. They are surrounded by a capsule of connective tissue containing a chronic inflammatory cell infiltrate and islands of odontogenic epithelium [5].

Anomalies of dental and facial development may be correlated with cancer treatment due to the lack of specificity of antineoplastic therapies such as chemotherapy and radiotherapy [6, 7], which do not differentiate between neoplastic cells and active healthy cells [6, 8].

Thus, the objective of the present study was to report a case of cystic odontoma affecting a pediatric patient diagnosed previously with Hodgkin's lymphoma (HL) who was treated by combination therapy mainly consisting of cyclophosphamide and vincristine.

## 2. Case Presentation

A black 5-year-old male patient was referred to the Stomatology Service of the Institute of Science and Technology, São Paulo State University, due to a swelling on the right side of his face. Anamnesis revealed that the patient had been diagnosed at age of 2 years and 8 months with mixed-cellularity classical HL, which was positive for Epstein-Barr virus (EBV), affecting the right side of the neck. His medical history showed that he had received a combination of surgery, chemotherapy, and radiotherapy. The chemotherapy protocol used consisted of 8 cycles at 21-day intervals of a combination of 760 mg/day cyclophosphamide, 1 mg/day vincristine, 7 mg/day bleomycin, 128 mg/day etoposide, 15 mg/day adriamycin, 155 mg/day dacarbazine, and 25 mg/day prednisone. Radiotherapy consisted of a total dose of 2160 cGy fractionated into 12 applications of 180 cGy/day to the right cervical, supraclavicular, and infraclavicular fields and the upper mediastinum.

**Figure 1:**
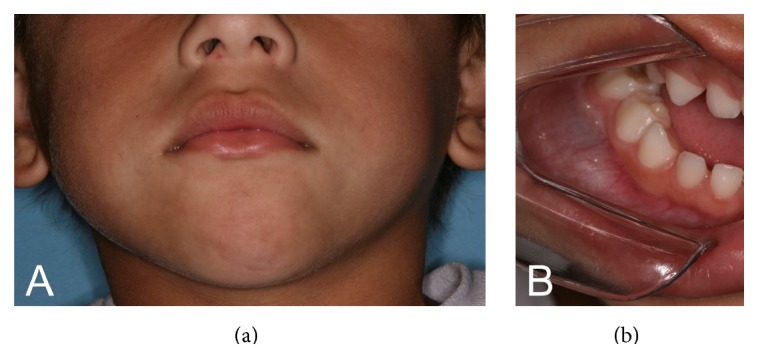
(a) Initial clinical appearance showing a swelling on the right side of the mandible. (b) Intraoral view.

**Figure 2:**
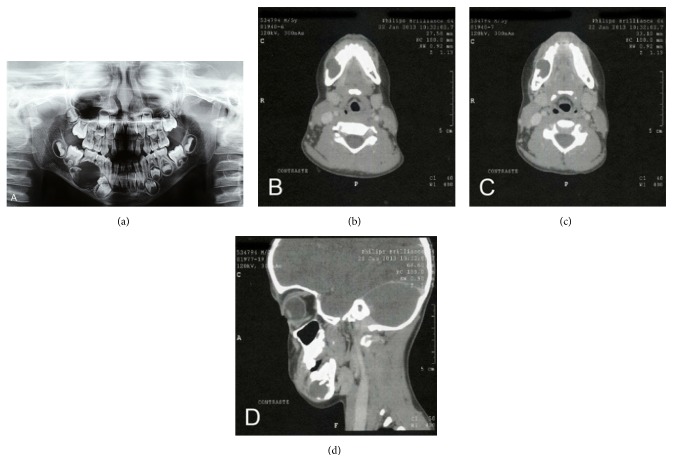
(a) Panoramic radiograph showing a well-defined radiolucent area associated with radiopaque material in the region of the mandibular premolars. (b and c) Computed tomography. Axial view: hypodense area containing well-defined hyperdense material. (d) Sagittal view.

Extraoral physical examination revealed a discrete swelling on the right side of the mandibular region (Figure 1(a)). Intraoral examination showed bulging of bluish color, asymptomatic and fluctuating upon palpation in the mandibular right molar region (Figure 1(b)). Panoramic radiography revealed a unilocular circumscribed radiolucent image associated with a radiopaque image of dental tissue-like density, causing reabsorption of the roots of the primary mandibular right first and second molars. There was also thinning of the ipsilateral mandibular basal cortical bone and delayed root development of the first permanent molars, as well as anomalous formation of the germ of the permanent maxillary left second premolar (Figure 2(a)).

**Figure 3:**
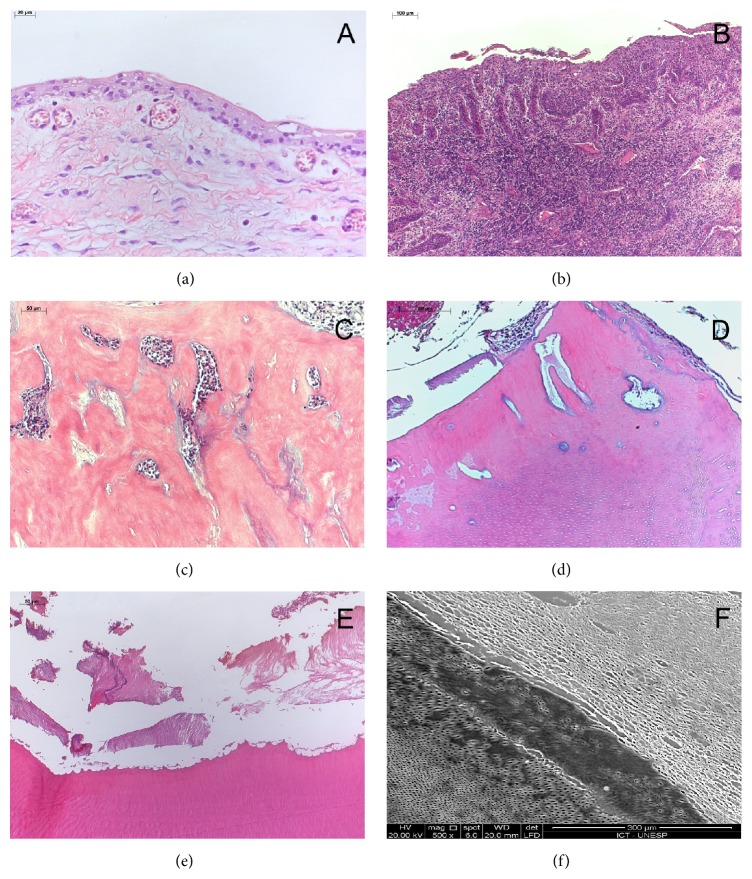
(a) Photomicrograph of the biopsy specimen showing a cystic lesion. Note the absence of inflammation (H&E stain). Surgical specimen. (b) Proliferative epithelium with an intense lymphoplasmacytic inflammatory infiltrate in the cystic capsule (H&E stain). (c and d) Fragments of disorganized calcified tissue composed of dentinoid and cementoid material, as well as focal areas of a basophilic substance resembling the enamel matrix. (e) Irregularities in the amelodentinal junction (H&E stain). (f) Hard tissue observed by scanning electron microscopy (magnification: 500x).

On the basis of the clinical differential diagnoses of giant cell lesion, odontogenic cyst, calcifying cystic odontogenic tumor, and ameloblastic fibrodentinoma/fibroodontoma, an incisional biopsy was performed. Microscopically, a cystic lesion lined with nonkeratinized atrophic stratified epithelium and supported by connective tissue without inflammation was observed (Figure 3(a)). Marsupialization was the management established and caregivers were instructed to irrigate it daily in order to reduce the lesion. However, they had difficulty in performing this daily procedure. After a 3-month follow-up, a new radiography showed reduction in the odontogenic cystic lesion and the patient was submitted to surgery for its complete removal.

Microscopic analysis of the surgical specimen revealed a proliferative epithelium exhibiting spongiosis and exocytosis, as well as an intense lymphoplasmacytic inflammatory infiltrate surrounded by granulation tissue in the cystic capsule (Figure 3(b)). Fragments of calcified tissue composed of cementoid and/or dentinoid material, as well as focal areas of a basophilic substance resembling the enamel matrix, supported by fibrocellular connective tissue and in close contact with hyperplastic epithelium, were observed at the periphery (Figures 3(c)–3(e)). The correlation of the clinical, radiographic, and microscopic findings was consistent with the diagnosis of complex odontoma associated with an odontogenic cyst (dentigerous cyst).

The calcified material was analyzed by scanning electron microscopy according to the modified protocol of Tay et al. [9]. Glass slides were metal sputtered with a SC7620 Mini Sputter Coater (Quorum Technologies Ltd., Ashford, United Kingdom) and observed under an Inspect S50 scanning electron microscope (Fei TM, Hillsboro, Oregon, USA) using a voltage of 20 kV and a mean working distance of 20 mm. The tissue exhibited irregularities in the amelodentinal junction, as well as disorganization of dentinal tubules. Areas of cementum-like material and pulp tissue were also observed (Figure 3(f)).

The patient has been in periodic monitoring and under dental treatment with pediatric dentist; after 36 months of follow-up he shows no signs of relapse, although he has some common side effects of chemotherapy, like premature apex formation in the first permanent molars and conical roots in mandibular incisors as well as anomalous formation of the germ of the permanent maxillary left second premolar and delayed development of the other second permanent molars.

## 3. Discussion

Hodgkin's lymphoma accounts for 6% of all childhood cancers. There are three distinct forms of HL: (a) the childhood form which occurs in children aged 14 years or younger; (b) the young adult form which affects individuals aged 15 to 34 years; and (c) the older adult form which commonly affects individuals aged 55 to 74 years. EBV positivity is more common among children younger than 10 years, is commonly observed in mixed-cellularity HL, and is almost never seen in lymphocyte-predominant HL. EBV serological status is not a prognostic factor for failure-free survival in pediatric patients with HL [10]. The mixed-cellularity subtype is the most common. The presence of EBV seems to be related to age and socioeconomic factors [10].

The rapidity of response to initial cycles of chemotherapy seems to be an important prognostic factor and is being used in the research setting to determine subsequent therapy. The present patient was diagnosed with mixed-cellularity EBV-positive classical HL, childhood form, and was successfully treated with surgery, radiotherapy, and chemotherapy.

Chemotherapeutic agents can cause enamel and dentin hypoplasia, conical roots, short apices, premature apex formation [6, 7, 11–15], anomalous teeth [16], and cysts [12, 17], as observed in the present case. Cyclophosphamide is considered to be cytostatic, acting on DNA and inhibiting cell division [7]. Animal studies have demonstrated that the damage caused by cyclophosphamide is limited to primitive mesenchymal cells and preodontoblasts, preventing ameloblast differentiation [18, 19] due to the absence of odontoblasts and to their inductive influence on epithelial cells of the inner layer of the enamel organ [20]. Furthermore, cells of the enamel organ, of the intermediate layer, and of the stellate reticulum exhibit vacuolization, cytoplasmic lamellar inclusion, nuclear disorganization, and necrosis when observed by scanning electron microscopy [19]. Necrosis may be the consequence of an autolytic process provoked by the chemical modification of cellular DNA bases by cyclophosphamide [19].

Some chemotherapeutic agents reduce the mitotic and secretory activity of odontoblasts and ameloblasts [8, 11], interfering with the formation of collagen fibrils, with the secretion of the dentin matrix [21], and with calcium transport in the ameloblasts [12]. In addition, vinca alkaloids (vincristine and vinblastine) destroy the brush border membrane of ameloblasts, preventing them from removing the protein content of the enamel matrix [22]. Furthermore, during the period of vincristine administration, there is an increase in the number and thickness of incremental lines in dentin [21, 23].

Chemotherapy can lead to the formation of osteodentin, which represents a niche or an irregularity in the amelodentinal junction that biochemically possesses a smaller quantity of the phosphorylated protein responsible for the nucleation of hydroxyapatite [22]. Abnormal osteodentin alters dentinogenesis and affects enamel mineralization, resulting in enamel hypoplasia over the defective dentin [22]. Dental abnormalities are related to the stage of tooth development, which is correlated with the age range of children at the beginning of chemotherapy as well as with the type, intensity, and frequency of the administered drug [7, 18]. These effects are potentiated when the cells are in a state of intense proliferation, as observed in patients younger than 5 years [12], and are mainly due to the use of drugs such as cyclophosphamide and vincristine [11, 14], as was the case in the present study.

The use of combined chemotherapy and radiotherapy in pediatric patients increases the risk of dental abnormalities [22]. Radiotherapy acts directly on odontoblasts, inhibiting their mitotic activity [15], and indirectly on the formation of enamel since it induces the formation of osteodentin replacing normal dentin [15, 24] by a mechanism similar to that observed with the use of chemotherapeutic agents [22]. The patient reported here had started chemotherapy at 3 years of age, a fact that might have contributed to the pathogenesis of the cystic complex odontoma since odontogenesis of the second lower premolar starts at about 2 and 1/2 years of age [25].

Odontomas associated with cystic lesions, as in the case of a dentigerous cyst, are uncommon [1, 3, 4]. Radiographically, this association appears as a mixed image containing radiolucent and radiopaque areas [3], with the differential diagnosis including calcifying cystic odontogenic tumors and ameloblastic fibroodontomas. Microscopically, cystic odontomas have characteristics of both dentigerous cysts and of complex odontomas [5], as observed in the present case.

## 4. Conclusion

Pediatric oncology patients who received antineoplastic treatment during the phase of odontogenesis may develop dental structure defects such as hypoplastic dentin and enamel, conical roots, root shortening, premature apex formation, agenesis, and anomalous teeth. These defects may imply esthetic, occlusal, and functional disorders and dentists should be aware of these possible side effects. Therefore since chemotherapy can affect the growth and development of infant teeth, a relationship between chemotherapy-associated adverse events and cystic odontoma should be considerate.
